# Supplementary material for: Efficacy of the Cardiac Implantable Electronic Device Multisensory Triage-HF Algorithm in Heart Failure Care: A Real-World Clinical Experience
Source: Sensors (Basel). 2024 Jun 5;24(11):3664. doi: 10.3390/s24113664 (PMC11175325; doi:10.3390/s24113664)
Supplement: Supplementary file 1 [file sensors-24-03664-s001.zip › sensors-2919090-supplementary.pdf]

sensors

MDPI

Supplementary Material

Contributing Sensory Data	Description	Key Insights
Thoracic Impedance	Plots daily averages of intrathoracic impedance against reference trend	Impedance decreases ~15 days before symptoms and 18 days before hospitalization
OptiVol Fluid Index	Measures spread between daily averages and reference trend	Shows fluid accumulation differences between daily and reference impedance
AT/AF Burden	Plots total time spent in AT/AF daily	AT episodes can trigger pulmonary congestion/volume overload
Ventricular Rate During AT/AF	Displays daily average and max ventricular rates during AT/AF	Poor rate control with atrial tachyarrhythmias linked to earlier hospitalizations
Patient Activity	Tracks activity counts sensed each minute via device accelerometer	Activity levels decrease before heart failure hospitalization
Heart Rate Variability (HRV)	Plots median atrial heart rate variability daily	Low HRV linked to higher hospitalization risk; high HRV indicates lower risk
Percent Pacing Per Day	Shows daily percentage of atrial and ventricular pacing	Important for optimizing CRT; poor rate control in AF can compromise CRT
Shocks Per Day	Counts days with defibrillation, cardioversion, or atrial shock therapy	
Treated VT/VF Episodes Per Day	Records daily total of spontaneous VT and VF episodes	

Supplementary Figure S1: Trended diagnostic data available in the Medtronic Heart Failure Management Report. *HF: Heart Failure, CRT: Cardiac Resynchronization Therapy, AT: Atrial Tachycardia, AF: Atrial Fibrillation, HRV: Heart Rate Variability, VT: Ventricular Tachycardia, VF: Ventricular Fibrillation.*

The Heart Failure Questionnaire	
Shortness of breath last night<br>Shortness of breath during the past 24 hours<br>Edema in the legs or abdomen<br>Feeling unwell during the past 24 hours<br>Increased Liquid oral intake during the past 24 hours<br><br>Excessive salt intake during the past 24 hours<br>Dizziness or lightheadedness<br>Dizziness or lightheadedness when standing up<br>Dizziness or lightheadedness when walking<br>Decreased intake during past 24 hours<br>Diarrhea or vomiting during past 24 hours	
≥ 2kg weight gain in 2 days<br>Higher blood pressure than normal	
New arrhythmia	

Supplementary Figure S2. The heart failure questionnaire.
